# Proceedings of the Thirteenth Annual UT- KBRIN Bioinformatics Summit 2014

**DOI:** 10.1186/1471-2105-15-S10-I1

**Published:** 2014-09-26

**Authors:** Eric C Rouchka, Julia H Chariker

**Affiliations:** 1 Department of Computer Engineering and Computer Science, University of Louisville, Duthie Center for Engineering, Louisville, KY 40292, USA; 2 Department of Psychological and Brain Sciences, University of Louisville, Louisville, KY 40292, USA

## 

The University of Tennessee (UT) and the Kentucky Biomedical Research Infrastructure Network (KBRIN) have collaborated over the past thirteen years to share research and educational expertise in bioinformatics. One result is an annual regional summit for researchers, educators and students interested in bioinformatics. The Thirteenth Annual UT- KBRIN Bioinformatics Summit was held at Lake Barkley State Park in Cadiz, Kentucky on April 11-13, 2014. A total of 172 participants pre-registered, with 104 from Tennessee and 61 from Kentucky. Among the registrants were 69 faculty, 45 students, 38 staff, and 20 postdocs. The conference program consisted of a workshop on Cytoscape and three days of presentations broken into plenary sessions on Genetic Variation and Mutational Analysis, Genomics, and P4 Systems Biology. Nine short talks were selected from 51 submitted poster abstracts.

## Friday workshops

Keiichiro Ono (UCSD) opened the Summit with the workshop “PART I: Introduction to Cytoscape: an Open Source Platform for Biological Network Data Analysis and Visualization.” He provided a description of the basic UI, methods for importing and exporting data, and methods for highlighting and exploring information in a biological network [1-3]. Keiichiro discussed methods for importing publicly available databases with a network structure, such as STRING [4], IntAct [5], BioGRID [6], ChEMBL [7], KEGG [8], Reactome [9], WikiPathways [10], and PathwayCommons [11].

Keiichiro presented a number of commands for simplifying networks, including selecting first neighbors of a node, creation of a subgraph, and filtering based on node and edge attributes. He also demonstrated tools for highlighting information in a network, including changing the visual properties of nodes and edges.

The second workshop, “PART II: Hands-On: Biological Network Analysis and Visualization with Cytoscape,” focused on advanced topics, including effective visualization techniques and external network analysis. Keiichiro provided a description of the network layouts available, such as hierarchical, force-directed, circular, and manual. In addition, he provided guidelines for choosing the most effective visual and spatial properties.

## Session I: genetic variation and mutational analysis

Hannah Carter (UCSD) began the formal program with “Identifying cancer drivers from high-throughput sequencing data” which focused on single nucleotide somatic mutations occurring in cancer samples. The goal was to determine which mutations are “drivers” contributing to tumorogenesis by altering protein activity, and which are “passengers” in the process. This is complicated since genomic abnormalities from 50 cancer types shows only a small number of mutations drive tumor progression [12]. She closed by describing the Cancer-Specific High-Throughput Annotation of Somatic Mutations (CHASM) approach for prioritizing driver SNVs [13].

Travis Burleson (Affymetrix) concluded the Friday evening session with “Mapping changes in the transcriptome: A primer into alternative splicing and how the Affymetrix TAC 2.0 software estimates these events.” Affymetrix has recently developed an array, HTAv2, for analysing alternative splicing in humans. Travis discussed in detail both the chip platform as well as the Transcript Analysis Console (TAC).

Saturday morning began with the final presentation in Genetic Variation and Mutational Analysis. Steve Horvath (UCLA) presented “Empirical evaluation of prediction- and correlation network methods applied to genomic data.” The bulk of the presentation focused on weighted gene co-expression network analysis (WGCNA) for identifying clusters or modules of highly correlated genes [14]. One of the key aspects of WGCNA is that it identifies a single gene within each module, called an eigengene, which best represents the pattern of gene expression found across all genes in the module. Dr. Horvath pointed out that identification of intramodular hub genes provides more meaningful information about a biological network than identification of hubs within the entire network. He also discussed a prediction method called the random generalized linear model (RGLM) [15] which is a combination of a random forest [16,17] and a forward regression model.

## Session II: genomics

Dr. Alistair Forrest (RIKEN Center for Life Science Technologies) opened the Genomics session on Saturday morning with “FANTOM5 – a mammalian promoter level analysis.” The FANTOM (Functional ANnoTation Of the Mammalian genome) project is an international research consortium established in 2000 with an initial goal of annotating over 20,000 cDNAs sequenced as part of the RIKEN Mouse Gene Encyclopedia Project [18]. The aim of FANTOM 5 was to generate a map of most human promoters as well as to generate comparative transcriptional network models of each cellular state.

Dr. Forrest focused on the results presented in three manuscripts published just prior to the Summit. The first, “A promoter level mammalian expression atlas” [19] reveals the results of CAGE across 975 human and 399 mouse samples, including primary cells and cancer cell lines. The second, “An atlas of active enhancers across human cell types and tissues” [20], identified 43,011 enhancer candidates within 432 primary cell samples, 135 tissue samples, and 241 cell line samples, all from humans, using overlaid ChIP-seq and CAGE sequencing results within transcription start sites. The third paper, “Interactive Visualization and analysis of large-scale NGS data-sets using ZENBU,” focused on the visualization toolkit ZENBU created for analysing large-scale sequencing datasets [21].

John Zhang (Life Technologies) closed the genomics session on Saturday evening with a discussion of the Ion Torrent PGM^TM^ and Proton^TM^ instruments. John discussed a number of applications available on these machines, with a specific focus on the Ion AmpliSeq^TM^ panels for detecting single nucleotide variants (SNVs) and insertions and deletions (indels). John focused on demonstrating the Ion Reporter^TM^ software for bioinformatics analysis [22].

## Session III: P4 systems biology

Nathan Price (The Institute for Systems Biology) kicked off the P4 Systems Biology session on Sunday morning with “Harnessing omics data for biological and medical discovery.” Dr. Price discussed several projects focused on systems approaches to integrating omics data in order to approach medicine from a personalized perspective. Included in his discussion was a project looking at honey bees as a model organism for examining social behaviors such as aggression, maturation, and foraging [23]. Dr. Price also presented a project that illustrates the usefulness of the Allen Brain Atlas [24] for characterization of cell type-specific genes [25] in which it was discovered that positional clustering of 170 neuron-specific genes reproduced the brain’s spatial structure. He also described SNAPR, a pipeline for RNA-seq alignment and analysis [26]. SNAPR contains a very efficient alignment algorithm, running approximately 25 times faster than TopHat [27] and Bowtie [28]. The final topic focused on P4 (predictive, preventative, personalized, and participatory) medicine [29]. He described in detail two projects currently underway at The Institute for Systems Biology – the Pioneer 100 project and the ISB 100K Wellness Project [30,31].

Joel Dudley (Icahn School of Medicine at Mount Sinai) concluded the invited speaker portion of the summit with “Integrating the digital universe of information for better models of disease and drug response.” Dr. Dudley’s presentation focused on medical discovery through big data bioinformatics. He described the BioMe Biobank project at Mount Sinai, which combines big data and personalized medicine with the goal of collecting samples from 100,000 donors. Dr. Dudley emphasized the importance of using publicly available data for a variety of research questions. In addition, he discussed the development of immune-pharmacology networks based on a variety of high-dimensional data [32].

## Posters and short talks

The poster session was held on day two. Fifty-one posters were on display from a variety of different research areas. A number of posters were also selected for short talks. These included “A new set-valued system identification approach to identifying rare genetic variants for ordered categorical phenotype” (Guolian Kang, St. Jude Children’s Research Hospital); “Building a knowledge base to assist clinical decision-making using the Pediatric Research Database (PRD) and machine learning: A case study on pediatric asthma patients” (Naga Nagisetty, Le Bonheur Children’s Hospital); “Differential isoform expression provides comprehensive stage-dependent signatures in cancer” (Qi Liu, Vanderbilt University); “Piecing the puzzle together: a revisit to transcript reconstruction problem in RNA-seq” (Yan Huang, University of Kentucky); “An island-based approach for differential expression analysis” (Abdallah Eteleeb, University of Louisville); “Evaluating four major algorithms for identifying differential regulators in condition-specific transcriptional responses” (Hui Yu, Vanderbilt University); “Development of sparse Bayesian multinomial generalized linear model for multi-class prediction” (Behrouz Madahian, University of Memphis); “Transcriptome profile of OVCAR3 cisplatin-resistant ovarian cancer cell line” (Sammed Madape, Meharry Medical College); and “Development of large-scale metabolite identification methods for metabolomics” (Hunter Moseley, University of Kentucky). For full author lists and abstracts see the rest of the supplement.

## Future plans

The 2015 Bioinformatics summit will return to Tennessee in the spring of 2015. Potential areas include current trends in molecular biology, applications of next-generation sequencing, and systems biology.

## Competing interests

The authors declare that they have no competing interests.

## Authors’ contributions

ECR served on the program committee for the UT-KBRIN Bioinformatics Summit and collected the final abstracts from selected authors. ECR and JHC contributed equally to the writing of the meeting summary.

## Declarations

Publication of this supplement was funded, in part, by the National Institutes of Health (NIH) and the National Institute of General Medical Sciences (NIGMS) under grant P20GM103436. The article contents are solely the responsibility of the authors and do not represent the official views of NIH or NIGMS.
